# Neutron Capture Radiography: Neutron Capture Radiography:a technique for isotopic labelling and analytical imaging with a few stable isotopes

**DOI:** 10.1100/tsw.2006.123

**Published:** 2006-06-19

**Authors:** Michel Thellier, Camille Ripoll

**Affiliations:** Laboratoire AMMIS, CNRS (FRE 2829), Faculté des Sciences de l'Université de Rouen, F-76821 Mont-Saint-Aignan Cedex, France

**Keywords:** lithium, boron, nitrogen, biological samples, physical methods of labelling and imaging

## Abstract

NCR (neutron capture radiography) may be used successfully for the imaging of one of the stable isotopes of a few chemical elements (especially Li and B, possibly also N, O, and others) and for labelling experiments using these stable isotopes. Other physical techniques compete with NCR. However, NCR can remain extremely useful in a certain number of cases, because it is usually more easily done and is less expensive than the other techniques.

## INTRODUCTION

To study the biological role of atoms and molecules requires the ability to detect them and, even more, to map them quantitatively in the biological systems under study (for a recent example, see Kyriasis and Tzaphlidou[1]). It is also important to be able to label them with appropriate tracers in order to study their transport and metabolism. The use of radioactive isotopes has been essential in such studies. Countless publications have dealt with radioisotope labelling and radioautographic techniques have long been brought to an extreme degree of sophistication[2,3,4,5,6]. There are, however, a few elements of biological relevance that do not possess any radioactive isotope with a half-life appropriate for isotopic labelling and radioautography. In these cases, it is sometimes possible to make use of a technique based on thermal neutron–induced nuclear reactions with nuclides whose cross-section for thermal neutron capture is sufficiently high. This technique, which was formerly given a variety of different names such as autoradiography by neutrons[7,8,9], nuclear reaction radiography[10], neutron-induced autoradiography[11], alphaneutronography[12], neutronography[13], alphagraphy[14], or microneutronography[15], is preferably called now neutron capture radiography (NCR)[16].

## GENERAL CONDITIONS OF APPLICATION OF THE NCR TECHNIQUE

A typical nuclear reaction is written X(x, y)Y. This means that the interaction of a radiation, x (a thermal neutron in our present case), with a nuclide, X, produces another nuclide, Y, with the emission of one or several rays, e.g., a γ-ray, a proton, or an α particle (an α particle being a helium nucleus, $^{4}He$). The main nuclear reactions of interest are (n, p) or (n, α) reactions, or else neutron-induced fissions of heavy nuclides (see Table 1). The first experiments in which a neutron-induced nuclear reaction was used for the analytical imaging of a nuclide in samples (including biological samples) took place in the 1950s[7,8,9,10,18,19]. The biological applications of NCR have been mainly with $^{10}B$ and $^{6}Li$, both because of the large cross-sections of these nuclides for thermal-neutron capture and because $^{10}B$- and $^{11}B$-enriched boron and $^{6}Li$- and $^{7}Li$-enriched lithium are commercially available.

**TABLE 1**
**Characteristics of the Main Nuclides Involved in Capture Reactions of Thermal Neutrons**

| Nuclide | Mean Natural Isotopic Abundance (% at/at) | Nuclear Reaction | Thermal Neutron Cross-Section (barn) |
|---|---|---|---|
| $^{6}Li$ | 7.5 | $^{6}Li(n, \alpha)^{3}H$ | 941.4 |
| $^{10}B$ | 19.9 | $^{10}B(n, \alpha)^{7}Li$ | 3838.2 |
| $^{14}N$ | 99.63 | $^{14}N(n, p)^{14}C$ | 1.83 |
| $^{2}D$ | 0.015 | $^{2}D(n, \gamma)^{3}H$ | 0.051 |
| $^{17}O$ | 0.038 | $^{17}O(n, \alpha)^{14}C$ | 0.24 |
| $^{32}S$ | 95.02 | $^{32}S(n, \alpha)^{29}Si$ | 0.004 |
| $^{33}S$ | 0.75 | $^{33}S(n, p)^{33}P$ | 0.002 |
| $^{33}S$ | 0.75 | $^{33}S(n, \alpha)^{30}Si$ | 0.14 |
| $^{35}Cl$ | 75.77 | $^{35}Cl(n, p)^{35}S$ | 0.48 |
| $^{39}K$ | 93.26 | $^{39}K(n, \alpha)^{36}Cl$ | 0.0043 |
| $^{40}K$ | 0.0117 | $^{40}K(n, p)^{40}Ar$ | 4.4 |
| $^{40}K$ | 0.0117 | $^{40}K(n, \alpha)^{37}Cl$ | 0.39 |
| $^{40}Ca$ | 96.94 | $^{40}Ca(n, \alpha)^{37}Ar$ | 0.002 |
| $^{235}U$ | 0.72 | $^{235}U(n, f)$ | 583 |

Symbols: $^{2}D = {}^{2}H$ = deuterium, n = neutron, p = proton, α = α particle, f = fission (NB: the fission products are variable and, thus, have not been indicated in this table). Data taken from the "Chart of the Nuclides"[17]. NCR is usually utilised for the detection of one or several of the first three nuclides ($^{6}Li$, $^{10}B$, and $^{14}N$), while the other nuclides are mainly involved in background problems.

The biological specimens used in the NCR technique may be histological sections, cell cultures, gelled droplets, etc. For the measurements, the specimens have to be dehydrated or they are sometimes ashed into "spodograms" (i.e., preserving the general structure of the tissues)[20,21]. When studying a mobile substance (such as the ion $Li^{+}$), one has to use preparation methods (e.g., cryomethods) that limit the risk of disturbing the natural distribution of this substance. The dehydrated or ashed sample is laid flat on an appropriate detecting film, then the whole arrangement (sample plus detecting film) is exposed to neutron irradiation. The impact of the ionizing particles produced by the nuclear reactions creates latent tracks in the detector. These latent tracks are "revealed" into visible tracks using an appropriate chemical treatment and the distribution of the tracks in the detector is representative of the distribution of the nuclide under study in the sample.

## TECHNICAL DETAILS

### Detectors and Lateral Resolution

In the first applications of the NCR technique, the tracks of the ionising particles created by the neutron nuclear reactions were recorded into "nuclear" photographic emulsions. They were developed according to the usual photographic procedure. In some cases, these photographic detectors were prepared extemporaneously by use of melted nuclear emulsions. By optimising all the steps of the operation[22], it was even possible to record the trajectory of each particle within the photographic emulsion and thus to identify this particle. However, it became rapidly apparent that "homogeneous" (also termed "solid-state") detectors (e.g., foils of high polymers such as cellulose nitrate) were much more practical and easy to handle than photographic emulsions to detect impact by ionising particles such as protons and α-rays[11,23,24,25,26,27]. When detectors made of a thin film of a polycarbonate or of cellulose nitrate became commercially available, most NCR studies were carried out using these detectors. In the cellulose nitrate films (or other solid-state detectors), the damage trails left by the particles induced by the nuclear reactions are detected by use of chemical etchants (such as solutions of NaOH or KOH) that dissolve the damaged material more rapidly than the bulk of the detector[27,28]: this makes the tracks appear as fine holes visible in optic microscopy. The lateral resolution of the technique can be estimated by the approximate size of the tracks in the detector. From the micrographs in the paper of Lelental[27] the lateral resolution may be calculated to be of the order of a few micrometers. For more detail about homogeneous detectors, see Thellier et al.[29].

## Neutron Irradiation

Among various other possibilities[13,30], nuclear reactors provide the most usual sources of neutrons for NCR applications, sometimes using a position in the reactor hall that delivers "cold" neutrons (i.e., neutrons with energy ≤ 10 meV[31,32]). The integrated neutron fluxes were usually in the range of $10^{10}$ to $10^{13}$ neutrons·cm$^{-2}$. $^{252}$Cf neutron sources were used sometimes[33,34] under conditions when no nuclear reactor was easily available. In that case, the integrated neutron flux was of the order of $10^{9}$ neutrons·cm$^{-2}$.

## Quantitative Evaluations

From the quantitative point of view, the local densities of tracks on a detector are evaluated by counting the number of tracks per field area of the microscope or by use of an automatic image analyser. With gelled droplets or other homogeneous samples, it is relatively straightforward to calculate the concentration of the nuclide under study in the sample from the density of tracks on the detector by use of an appropriate calibration curve determined in a preliminary experiment[27,35]. With nonhomogeneous specimens (which is usually the case with biological samples), the problem of the evaluation of local concentrations in the specimen is much more arduous. It can be approached, however, via an appropriate modelling of the specimen structure[36]. Several different cases have been considered: (1) one or several types of particles involved; (2) initial energy of the particles below, or above the "upper threshold of detection"; (3) possible non-negligible abrasion of the detectors during etching; or (4) contribution of background tracks originating from the detectors themselves. In all these cases, the modelling consisted of evaluating the probability that a particle, created by the nuclear reaction under consideration, leaves a track in the detector; the value of this probability appeared to depend on a calibration parameter, the value of which was estimated in a preliminary experiment with a calibration sample.

## Discrimination

When several different nuclides present in the sample are engaged into nuclear reactions with neutrons, appropriate discrimination of the corresponding tracks in the detector has to be carried out. The problem is especially important for the three nuclides $^{6}$Li, $^{10}$B, and/or $^{14}$N, which are usually the subject of NCR detection. It is easy to discriminate the small proton tracks produced by $^{14}$N from the big α, $^{3}$H and $^{7}$Li tracks produced by $^{6}$Li or $^{10}$B (Fig. 1). As a consequence, it is possible to refer local concentrations of $^{6}$Li or $^{10}$B in

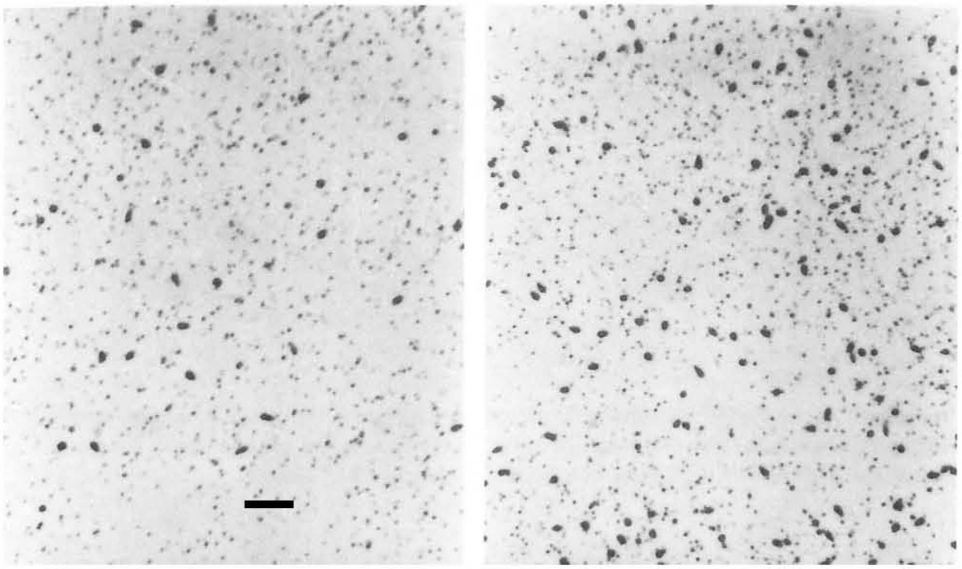

**FIGURE 1.** Discrimination of $^{4}\alpha/^{7}Li$ tracks of $^{10}B$ and of proton tracks of $^{14}N$, in a cellulose-nitrate detector after neutron irradiation and detector etching. NCR images of a tissue sample poor (left) and richer (right) in boron. The big, dark tracks originating from $^{10}B$ are easily discriminated from the small, grey tracks originating from $^{14}N$. Bar = 10 µm. Modified from Laurent-Pettersson et al.[37].

a biological tissue to the corresponding local concentrations of nitrogen in this tissue. Discrimination of the tracks produced by $^{6}Li$ and $^{10}B$ can be based on the range of the particles created in the nuclear reactions. Ranges (expressed in micrometers in water) are ≤10 for the α and $^{7}Li$ particles issuing from $^{10}B$, and 11 and 45 for the α and $^{3}H$ particles from $^{6}Li$; therefore it is easy to get rid of the tracks issuing from $^{10}B$ and keep only the $^{3}H$ tracks from $^{6}Li$, by inserting an appropriate shield between sample and detector. Double labelling ($^{6}Li/^{10}B$) can thus be envisaged.

## Background

An appreciable advantage of cellulose-nitrate detectors compared with photographic emulsions is that they are insensitive to impact by β- and γ-rays. However, background tracks may be produced by nuclear reactions with the few rapid neutrons that inevitably accompany the thermal neutrons in the neutron fluxes of a conventional nuclear reactor. Examples of nuclear reactions with rapid neutrons are $^{14}N(n, \alpha)^{11}B$, $^{16}O(n, \alpha)^{13}C$, $^{14}N(n, ^{3}H)^{12}C$, $^{12}C(n, \alpha)^{9}Be$; these reactions occur with neutron energies (in MeV) above 0.2, 2.4, 4.3, and 6.2, respectively, and their cross-sections (in barn) are 0.15, 0.10, 0.10, and 0.02[38]. Given the relatively small values of these cross-sections and the fact that the proportion of rapid neutrons in the neutron flux of a reactor is usually low, the background originating from the reactions with rapid neutrons is usually reasonably low and its value can be evaluated and deduced from the measurements[38]. Moreover, this contribution to the background can be almost totally eliminated by using cold neutrons (energy ≤ 10 meV) instead of thermal neutrons (same energy spectrum as in a gas at normal temperature). Another contribution to the background originates from the nuclear reactions with thermal neutrons of those nuclides indicated in Table 1 that are present in the sample and/or in the cellulose-nitrate detectors. Given the usually low values of their cross-sections and/or their natural isotopic abundance, this contribution to the background is negligible when the integrated flux of neutrons is not too high, but it will eventually saturate the detectors at high neutron fluxes. This is the reason for not using integrated neutron fluxes higher than $10^{14}$ neutrons·cm$^{-2}$.

## Limits of Detection

By using a mass-spectrometric detection of the $^3H$ and $^4He$ (α particles) produced in the nuclear reactions on $^{10}B$ and $^6Li$, Clarke et al.[38] have announced a limit of detection better than $10^{-8}$ g·g$^{-1}$ for analysis of boron and lithium at ultratrace concentrations in biological samples. Using homogeneous samples (phosphate-buffered solutions of $^{10}B$-boric acid containing about 1 mg·ml$^{-1}$ protein) and the classical cellulose nitrate detectors, the limit of detection of $^{10}B$ was found to be significantly better than $10^{-6}$ g·g$^{-1}$ (close to 0.1 µg·g$^{-1}$)[35]. Since the cross-section for the reaction of thermal neutrons with $^{10}B$ is approximately four times as large as that with $^6Li$ (see Table 1), this means that the detection limit for $^6Li$ is also better than $10^{-6}$ g·g$^{-1}$.

## APPLICATION OF NCR TO LITHIUM STUDIES

Although lithium is not usually considered an essential element for life, the addition of lithium has very specific effects on all types of living beings, from viruses to humans[39], including beneficial medical treatments[40] and possible adverse effects[41].

Lithium localisation and transport in the brain of lithium-treated animals has been the subject of many studies, in relation with the medical treatment of manic-depressive psychosis in humans[40]. A clear-cut regionalisation of lithium was observed within the brain of Li-treated, normal adult animals[42,43,44,45]; the lithium concentrations were especially high in the neocortex, the hippocampus, the striatum, and the granular layer of the cerebellum, whereas the corpus callosum and the thalamus were much less concentrated in lithium (Fig. 2). By contrast, the distribution of lithium in the brain of mouse embryos[47,48] or of "quaking" dysmyelineting mutants[49] was almost homogeneous (not shown), which suggests that the regionalisation of lithium in the brain is somehow related with neuron myelination. The isotopic exchange of lithium in various brain substructures was studied with adult mice previously equilibrated with $^6Li$ then transferred to $^7Li$ at constant concentration of total lithium (plasma concentration of total lithium kept close to 0.28 m*M*)[50]. At any moment, the isotopic abundances of $^6Li$ (ratio of $^6Li$ to total lithium, at/at) in the different brain areas were not significantly different from one another. Moreover, the time courses of the isotopic abundance of $^6Li$ in the brain and in the plasma, which were both fitted by the composition of two exponential terms, were not significantly different from each other at the precision of the experiments. This means that the isotopic equilibration of lithium between the different brain substructures and the plasma is almost instantaneous.

The NCR technique was also applied to the localisation of lithium in various tissues of the mouse embryos[47,48], in relation with the well-known teratogenic effect of lithium[51]. Lithium was especially concentrated in the bones, the heart, the eyes, and some endocrine glands (hypophysis, thyroid) of embryos borne by lithium-treated mice, while their liver, stomach, and lungs were significantly less concentrated. The high lithium concentrations in the bones do not seem to be associated with specific malformations of the developing bones. On the contrary, the high lithium content of the heart observed in the mouse embryos might be related to cardiac malformations[52] occurring in children born from lithium-treated women. Similarly, the high lithium contents occurring in embryonic brain in the early stages of pregnancy may be related to lithium-induced alterations of the differentiation of the neuroectoderm and of the optic vesicles in various vertebrates[53,54,55,56].

Subcellular localization of lithium was attempted using large-size, lithium-treated cells (glioma cells and oocytes). Although the experimental conditions were not optimal, it appeared likely that lithium tended to accumulate at, or close to, the plasma and nuclear membranes of the cells[57].

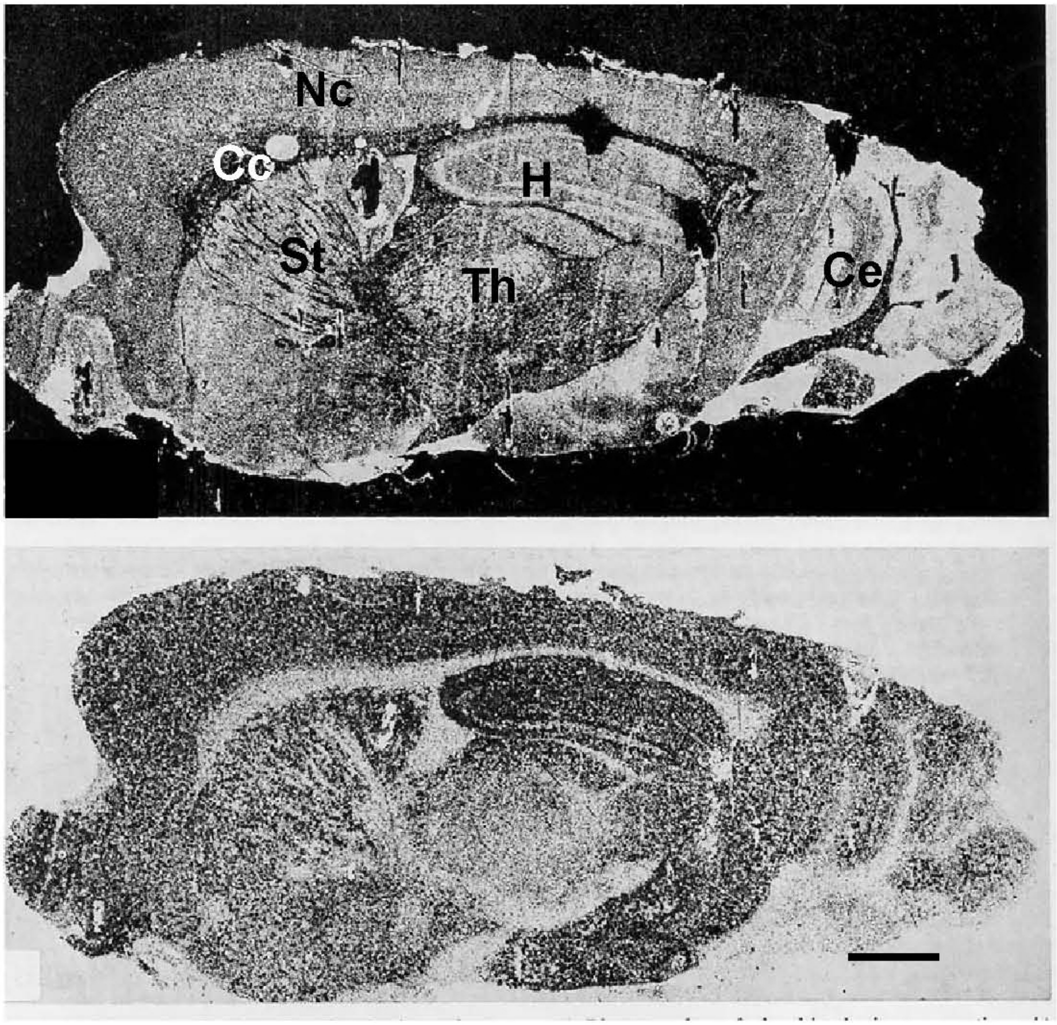

**FIGURE 2.** Lithium distribution in the brain of a lithium-treated mouse. Top: histological section of the mouse brain. Bottom: the corresponding NCR image of the distribution of lithium. The mice under study were given an intraperitoneal injection of $^{6}$LiCl (12 mmol·kg$^{-1}$) every 24 h during 3 days. After chloroform anaesthesia of the animals, the brains were sampled and cryofixed by dipping into liquid nitrogen. Sections (8–12 µm in thickness) were cut, laid on quartz supports, lyophilised, and covered with a cellulose-nitrate detector. After neutron irradiation (10$^{12}$ neutrons·cm$^{-2}$), the detectors were etched in 10% NaOH to make the distribution of the α/$^{3}$H tracks of $^{6}$Li appear as little black dots under photon-microscope observation. Hence, the darker a site in the NCR image, the richer in $^{6}$Li is the corresponding site in the brain tissue. The track density in the NCR images of $^{7}$Li-treated controls was close to zero. Symbols: Cc: corpus callosum, Ce: cerebellum, H: hippocampus, Nc: neocortex, St: striatum, Th: thalamus. Bar = 1 mm. Modified from Wissocq et al.[46].

## APPLICATION OF NCR TO BORON STUDIES

Boron is involved in many different biological processes, including enzyme degradation and transmembrane transport of sugars[58,59]. However, there are chiefly two cases when NCR was applied to boron studies[60]: (1) the imaging of boron-labelled molecules with a view to cancer treatment by BNCT (boron neutron capture therapy) and (2) the study of the boron nutrition of plants.

### NCR for BNCT

BNCT is based on using the same nuclear reaction, $^{10}$B(n, α)$^{7}$Li, which is used for the NCR imaging of boron. Briefly, a molecule that has an affinity for a given type of cancer cells is loaded with as many $^{10}$B

atoms as possible. When the cancer cells have absorbed the boron-loaded molecules, irradiation by thermal neutrons causes the very ionizing, short-range α and $^{7}Li$ particles that are emitted to be lethal for these cancer cells without significantly reaching the neighbouring, healthy cells. For the basic principles about BNCT see Sweet[61] and for more detail, see Barth et al.[30] and Sauerwein[62].

In this approach of cancer treatment, clearly it is decisive to make sure that the boron-loaded molecules invade massively the malignant cells without practically penetrating into the healthy ones. Labelling the boron-loaded compound with a radioactive isotope and determining the distribution of the radioactivity *in vivo* may be used to evaluate the efficacy of this compound for localizing selectively in the tumour cells[63]. As an alternative, it is also possible (especially when studying model systems) to prepare cell samples or histological sections of a tissue area of interest and examine the relative distribution of $^{10}B$ between tumour and healthy cells by NCR.

A lot of work has been devoted to find compounds that have a high specificity for malignant cells and that do not lose their specificity when loaded with boron[30]. A localization of these boron compounds in the cell nucleus would also be advantageous, since the heavy particles resulting from the neutron-capture reaction would deliver a greater proportion of their energy directly to the nucleus[30]. It is, thus, useful to try to carry out the subcellular localisation of boron in the cells of interest. Fig. 3 gives an example of the distribution of boron in human glioma cells grown for 24 h in the presence of 50 $mg{\cdot}L^{-1}$ BPA (paraborophenylalanine)[64]. Adapting the theoretical modelling meant for quantitative NCR measurements[36] to this particular problem, the natural concentration of boron in the glioma cells (nontreated controls) was evaluated to be close to 1 ppm (fresh weight), while there was extra accumulation of boron of approximately 10 and 4 ppm (fresh weight) in the cell nucleus and in the cytoplasm of the BPA-treated cells.

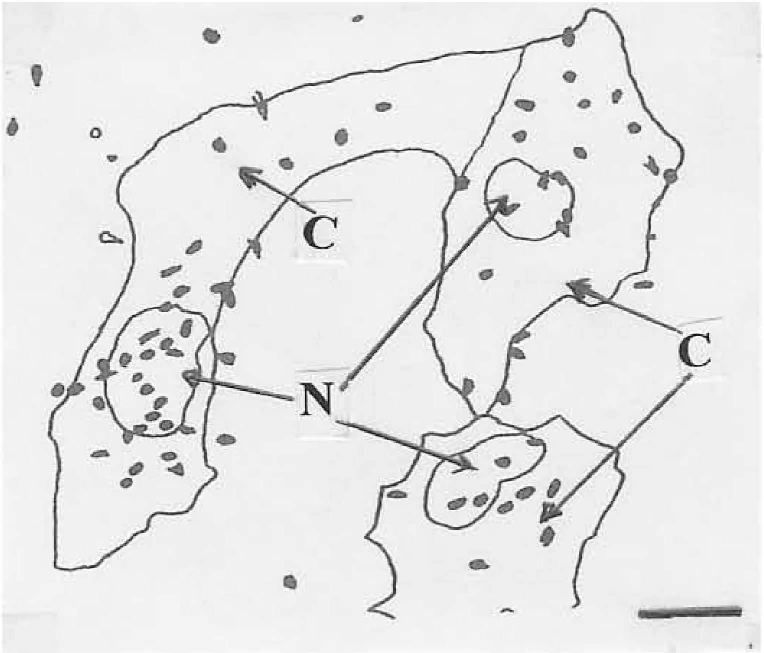

**FIGURE 3.** NCR imaging of boron in BPA-treated human glioma cells grown *in vitro*. The cells were grown in the presence of pieces of CR-39 polycarbonate sheets (Pershore Mouldings, Great Britain) to which they adhered. The cells were subjected to 24-h incubation in the presence of 50 $mg{\cdot}l^{-1}$ BPA. After washing, the CR-39 pieces bearing the cells were freeze dried and covered with another piece of CR-39 sheet, and the whole assemblies of the cells with the two CR-39 sheets were irradiated with an integrated flux of $5{\cdot}10^{12}$ $neutrons{\cdot}cm^{-2}$. The CR-39 pieces bearing the cells were separated from the CR-39 covers, coloured in hemalun and photographed (the contours of the cells and of the cell nuclei were then easily visible). The CR-39 covers, which are efficient detectors of the $\alpha/^{7}Li$ tracks induced by the interaction of neutrons with the $^{10}B$ atom nuclei, were etched in 6.25 *M* NaOH at 70°C for 1 h. The figure represents the superposition of the contours (cytoplasm and nucleus) of glioma cells redrawn from the photographs, with the corresponding distribution of $\alpha/^{7}Li$ tracks (the black dots on the figure) of boron on the CR-39 detector. Symbols: C = cytoplasm, N = cell nucleus. Bar = 20 µm. Modified from Laurent-Pettersson et al.[64].

## NCR for the Study of Physiological Boron in Plants

Boron is an essential micronutrient for plants[65,66,67], but excessive concentrations of boron are deleterious[68,69,70] (for reviews, see Shorrocks[71] and Nable *et al.*[72]). When plants are either totally deprived of boron or supplied with too massive amounts of boron, they die. Boron deficiency or excess occur frequently under natural conditions, which may affect crops very significantly[73]. In the case of a boron deficiency, a boron fertilizer may be supplied to the soil[73] or as foliar spray[74]. Our group has made an extensive use of NCR, with natural or $^{10}$B- and $^{11}$B-enriched boron, to study the regionalisation and the transport and exchanges of boron in plant samples.

In some cases, boron data were obtained by direct NCR imaging of boron in histological sections of plants. For instance[75], histological sections were cut in the hypocotyls of 10-day-old flax seedlings grown in a nutrient medium with 27.3 µ*M* boric acid (natural boron) and studied for determination of local boron concentrations by NCR. At the tissue level, the mean boron concentrations (mmol·kg$^{-1}$, dry weight) were highest in the phloem (almost 18), lowest in those tissues made up of cells with secondary walls (4.1 in the xylem and 2.8 in the differentiated fibres) and in the order of 9 to 13 in the other tissues (epidermis, cortical parenchyma, and medullar parenchyma). The evaluation of the boron concentrations in the cell walls was as indicated in Table 2. The primary walls appeared to be relatively rich in boron, especially in the xylem and the differentiated fibres, i.e., in those cells that possess a secondary wall, whereas the secondary walls in these same cells were almost deprived from boron. In another experiment with 15-day-old clover plants fed with 16 µ*M* boric acid, the boron concentrations (mmol·kg$^{-1}$, fresh weight) measured in cells of the leaf parenchyma were 7.64 in the cell wall, 0.35 in the cytoplasm, and almost zero in the vacuole. Moreover, by using plants fed with $^{10}$B- or $^{11}$B-enriched boric acid, it was shown that the cytoplasmic boron originated in almost equal amounts from the seed reserves and from absorption from the nutrient medium whereas in the cell wall the proportions were closer to one-fourth and three-fourths[76].

**TABLE 2**
**Boron Concentrations in the Wall of Hypocotyl Cells of 10-Day-Old Flax Seedlings Grown in a Nutrient Solution Containing 27.3 µ*M* Boric Acid**

| Plant Tissues | Boron Concentration in the Cell Wall (mmol·kg$^{-1}$, Dry Weight of Cell Wall) | |
|---|---|---|
| | Primary Wall | Secondary Wall |
| Epidermis | 8 | — |
| Cortical parenchyma | 16 | — |
| Differentiated fibres | 82 | 1 |
| Phloem | 28 | — |
| Xylem | 43 | 1 |
| Medullar parenchyma | 8 | — |

The concentration values were evaluated by combining NCR measurements, the modelling for quantitative evaluations from rough NCR data[36]), and a stereometric study of the plant tissues.

A totally different approach, based on measurements of the isotopic exchange $^{10}$B/$^{11}$B and on the theory of compartment analysis, was followed with intact samples of the water weed *Lemna minor* grown in the presence of 0.16 m*M* $^{10}$B-boric acid[77]. The experimental conditions and results are given in Table 3. In an experiment with roots of sunflower plants grown in the presence of 0.10 m*M* $^{11}$B-boric acid[79], it is not surprising that the boron concentrations in cell wall, cytoplasm, and vacuole (as evaluated by studying the

**TABLE 3**
**Subcellular Boron Data in *Lemna* Plants**

| Measured Quantity | Cell Structure | | Value |
|---|---|---|---|
| Boron concentrations (mM) | Wall | | 14.4 |
| | Cytoplasm | | 6.6 |
| | Vacuole | | 2.9 |
| Boron fluxes (pmol·cm$^{-2}$·h$^{-1}$) | Plasmalemma | Influx | 208 |
| | | Outflux | 198 |
| | Tonoplast | Influx | 11.7 |
| | | Outflux | 7.1 |

The plants were initially grown in a nutrient medium containing 0.16 m*M* $^{10}$B-boric acid. Their boron content at the moment of the experiments was 93.4 µmol·g$^{-1}$ (dry weight). At the beginning of the experiment, the plants were transferred to a medium identical in composition to their growth medium, but containing $^{11}$B-boric acid instead of $^{10}$B-boric acid. The isotopic exchange $^{11}$B/$^{10}$B was studied for 24 h. Boron concentrations in the various cell compartments and boron fluxes between compartments were calculated according to the classical approach of compartmental analysis. These data were expressed relative to the mass (dry weight) of total plants[77]. After a stereometric study of the *Lemna* cells[78], it was possible to express the boron concentrations in m*M* (related to the water content of the compartment under consideration) and the boron influx and outflux at the cell membranes (plasmalemma and tonoplast) in pmol·cm$^{-2}$·h$^{-1}$ (related to the unit of membrane surface).

isotopic exchange $^{11}$B/$^{10}$B via mass-spectrometry measurements) were different from those in the *Lemna* plants; however, the dimensionless values (referred to the boron content of the wall) were in the order of 1, 0.5, 0.14 in the sunflower roots, i.e., reasonably similar to those (1, 0.5, 0.2) that can be calculated with the *Lemna* plants from the data in Table 3. In the *Lemna* experiment, it appears also that not only subcellular boron concentrations, but also fluxes of boron between cell compartments, can be evaluated. Moreover, the study of the kinetics of uptake of $^{10}$B-boric acid by *Lemna* plants previously fed with $^{11}$B-boric acid has revealed the existence of a saturable component for external boric acid concentrations up to 0.5 m*M*, followed by an inhibitory effect at larger concentrations[80].

When a drop of a solution of $^{10}$B-boric acid was deposited on a leaf of clover seedlings previously fed with $^{11}$B-boric acid, a NCR study revealed that most of the leaf-absorbed $^{10}$B-boron remained immobilised at the level of the treated area. Only a small part of the foliar-absorbed boron migrated to the rest of the leaf, especially via the leaf nerves (Fig. 4), and to the other leaves of the plant (not shown). In a similar study, again with clover plants, it was revealed that more than 98% of the foliar-supplied boron remained immobilised at the point where it was applied to the leaves, while less than 2% distributed to the different parts of the plants was possibly remobilised from old parts to newly formed leaves and was useful to the growth of the treated plant[21]. At the histological level, the phloem was the tissue the richest in transported $^{10}$B on the day following the foliar treatment of the plants[81]. Boron thus appears to be very little mobile in clover plants. By contrast, the boron supplied by foliar spray was readily translocated within and out of treated leaves of coffee plants[34]. This particular mobility of boron in coffee plants might mean that coffee belongs to those species where boron is phloem mobile due to complexation with sorbitol[82].

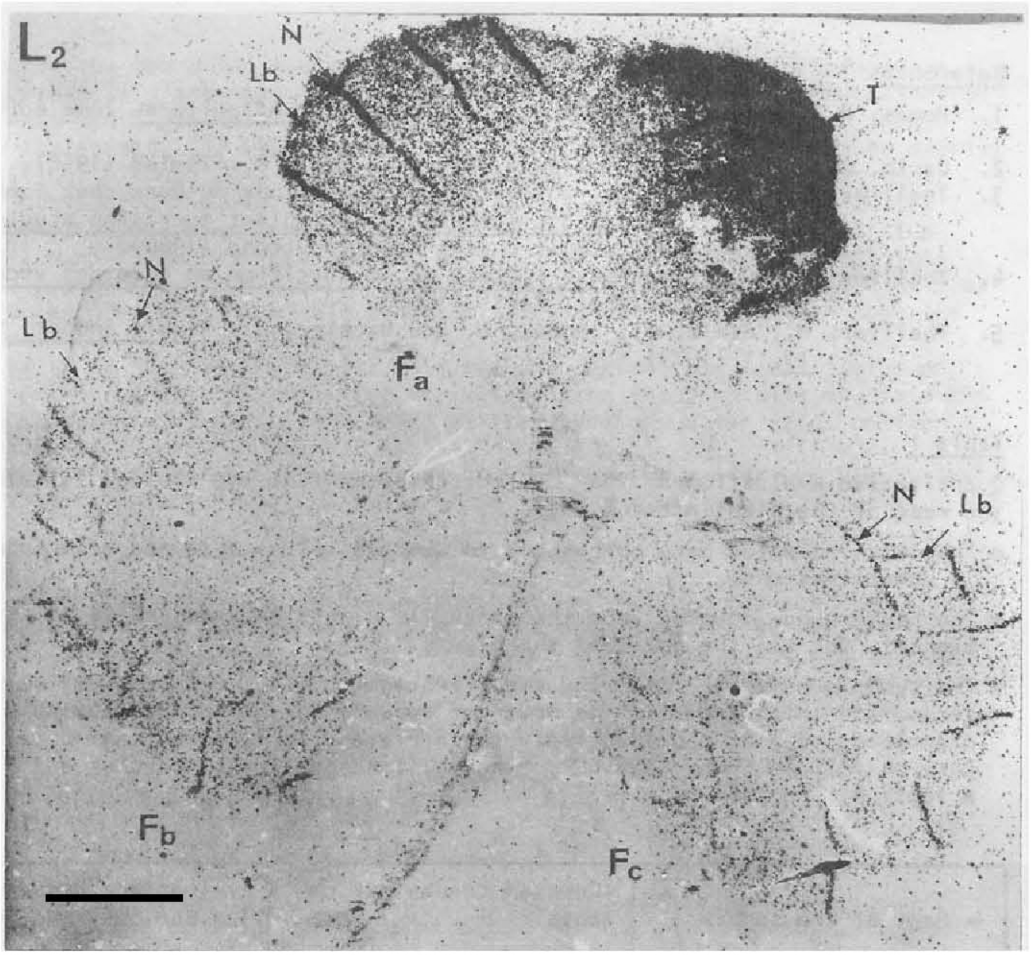

**FIGURE 4.** Foliar application of $^{10}B$-boric acid on seedlings of white clover previously fed with $^{11}B$-boric acid. When the seedlings were 20 days old (at that time they had two developed leaves plus their cotyledons), a 5-µl drop of a solution of $^{10}B$-boric acid (10 g·l$^{-1}$) was deposited on the extreme foliole of the second leave. Five days later (a third leaf had usually developed meanwhile), the boric acid possibly remaining unabsorbed on the treated leaf was washed out, the seedlings were displayed on a quartz blade where they were incinerated in such a way as to keep the ashes exactly in position (spodogram), and then the distribution of the absorbed $^{10}B$ was imaged by NCR. At this magnification, the impacts of the $^{4}\alpha/^{7}Li$ tracks created by the neutron nuclear reaction on $^{10}B$ appear as tiny black dots. With non-$^{10}B$-treated controls, only a small background of black dots was observed (not shown). Symbols: $L_2$ = second leaf of the seedling; $F_a$, $F_b$, and $F_c$ = the three folioles of leaf $L_2$; T = treated area (the leaf area where the $^{10}B$ drop was deposited); N = leaf nerve; Lb = leaf limb. Bar = 2 mm. Modified from Martini and Thellier[20].

## APPLICATION OF NCR TO THE STUDY OF VARIOUS OTHER ELEMENTS

In principle, any of the nuclides listed in Table 1 can be detected using NCR. The (n, p) nuclear reaction with $^{14}N$ was shown to be utilisable for the analytical imaging of natural nitrogen in biological samples[83,84]. A few measurements have also been carried out with $^{17}O$[32,85] and fissionable nuclei[86].

## DISCUSSION AND CONCLUSION

NCR today is challenged by other techniques for analytical imaging and for using stable isotopes in labelling experiments.

The efficacy of nuclear microprobe analysis (NMA)[87,88] and of secondary ion mass spectrometry (SIMS)[89,90] has been compared with that of NCR for nitrogen studies[91]. NMA consists of bombarding the specimen under study with a beam of protons (or other charged, light particles) and detecting all the atomic or nuclear events that are produced by the interaction of the bombarding particles with the sample atoms. In the SIMS method, the sample is bombarded with a beam of medium weight or heavy ions (primary ions) and the ions that are sputtered from the sample surface (secondary ions) are collected, sorted by mass spectrometry, and used for analytical and imaging purposes. Particular advantages of the NMA and SIMS techniques are that they can detect both isotopes of nitrogen, $^{14}N$ and $^{15}N$, and that their lateral resolution is 1–2 µm for NMA, of the order of 0.3 µm with classical SIMS machines and even better with the NanoSIMS50 instrument[92]. Moreover, NMA[93,94] and even more SIMS[95,96,97,98] have been utilized for nitrogen-isotope studies in biological material. SIMS may be used for $^{10}B$ and $^{11}B$ imaging in biological samples[99]; the sensitivity is not very good, but could be improved by postionisation[100]. In relation with BNCT, boron has been localised at the subcellular level by EELS (electron energy loss spectrometry)[101]. The SIRIMP (sputter-initiated resonance ionisation microprobe) and LARIMP (laser atomisation resonance ionisation microprobe) techniques are well suited for quantitative and ultrasensitive imaging of boron trace concentrations with subcellular resolution in biological tissue sections[100]. SIRIMP combines resonance ionisation with a high-energy pulsed ion beam and mass spectrometric detection, while LARIMP uses a laser pulse instead of an ion pulse for the atomisation process. SIMS has been used in $^{6}Li/^{7}Li$ isotopic exchange experiments for the determination of lithium concentrations in subcellular compartments and of lithium fluxes between these compartments[102]. Moreover, the SIMS method would be very well suited for the direct imaging of lithium isotopes in biological samples, especially because no interference by polyatomic ions may be expected at mass values equal to 6 and 7, although, to our knowledge, no such experiment has been carried out so far. Fissionable nuclei such as $^{226}Ra$ or uranium isotopes can be rapidly determined in solid samples by fusion with lithium metaborate and α-spectrometry[103].

In brief, very efficient physical methods can now compete with NCR for the imaging and the isotopic labelling of lithium, boron, and nitrogen in biological samples. Moreover, these other methods can usually detect and image the isotopes of many other elements than Li, B, and N (which also makes it possible to carry out multilabelling experiments). As a consequence, the NCR technique is progressively less utilized than it was during the second half of the 20th century. However there are at least two cases in which NCR may remain extremely useful: (1) for those researchers (especially in developing countries) for whom sophisticated physical techniques are not easily available or are too expensive and (2) for carrying out relatively simple and nonexpensive preliminary assays before going to more complex techniques.
